# New Appliances and Things Medical

**Published:** 1899-01-07

**Authors:** 


### NEW APPLIANCES AND THINGS MEDICAL.

[We shall be glad to receive, at our Office, 28 & 29, Southampton Street, Strand, London, W.C., from the manufacturers, specimens of all new preparations and appliances which may be brought out from time to time.]

### THE PATENT "AUTOMISER."

(The Automatic Fuel Economiser Company, 130, Queen Victoria Street, E. C.)

This is an arrangement for letting in warm air at the back of the fire and at the same time filling up that part of the fireplace combustion in which adds but little to the radiant heat emitted into the room. It is a hollow iron back placed in the position which is often filled up by a fire-brick. The hollow space serves as a hot air chamber, the air from which escapes through an opening in its lower part into the fire. It is claimed that the automisers give a bright fire and more heat from less fuel. There is no doubt they give a bright and cheerful fire, and as the sort of heat we want in the warming of a room is the radiant heat from the brightly burning part of the fire they ought to be economical. We have not given them a sufficiently prolonged comparative test to speak from experience, but there can be no doubt that for warming by radiation a small brightly burning fire such as is produced by the automisers is the best.

### GLYCEROL OF MALT.

(Price's Patent Candle Company, Limited, London and Liverpool.)

Glycerol of malt is, as its name implies, a combination of glycerine and malt extract. The combination is a happy one, both from the point of view of therapeusis and from that of elegance, for the glycerine acts as a powerful preservative of the digestive powers of malt extract, and is in itself a valuable food and intestinal lubricant, and possibly antiseptic. Further than this, the thick, ropy character of the malt extract is reduced to a comparatively thin consistency by the admixture, and, as far as taste is concerned, the agreeable sweetish taste of each is not impaired by the combination. This excellent digestive and food can act as an agreeable vehicle of such largely used drugs as creosote, cascara, squills, and ipecacuanha, and doubtless in this capacity it will find one of its most useful applications.

### CARNOSE: A SUBSTITUTE FOR MEAT.

(H. J. F. Crosby, The Tower Brewery, Grimsby.)

Carnose is a preparation made from yeast, and it is intended for use as a substitute for meat extracts. It is stated to contain a high percentage of albumenoids and substances corresponding with meat bases and peptones. The sample of Carnose submitted was of the nature of a jelly, of the colour of meat extract, and which dissolved easily in water. A spoonful dissolved in a cup of boiling water as directed gave a liquid resembling in some respects a similarly prepared solution of meat extract, but it had a decidedly bitter flavour. On analysis Carnose was found to contain 29·6 per cent. of organic matter, of which about one-half was nitrogenous matter, 5·28 per cent. mineral constituents, and 65·12 per cent. water. It has no doubt nutritive properties, and it becomes a matter of public taste as to its acceptance in place of meat extracts now so readily available. Certainly for vegetarians it has the advantage of having been prepared without the use of butcher's meat.

